# Supplementary material for: Selectivity and Ranking of Tight-Binding JAK-STAT Inhibitors Using Markovian Milestoning with Voronoi Tessellations
Source: J Chem Inf Model. 2023 Apr 6;63(8):2469–82. doi: 10.1021/acs.jcim.2c01589 (PMC10131228; doi:10.1021/acs.jcim.2c01589)
Supplement: Supplementary file 1 — ci2c01589_si_001.pdf [file ci2c01589_si_001.pdf]

Supporting Information: Selectivity and ranking of tight-binding JAK-STAT inhibitors using Markovian milestoning with Voronoi tessellations

Anupam Anand Ojha, Ambuj Srivastava, Lane William Votapka, and Rommie E. Amaro*

Department of Chemistry and Biochemistry, University of California San Diego, La Jolla, CA 92093, USA

E-mail: ramaro@ucsd.edu

Table S1: JAK2 and JAK3 residues within a cut-off distance of 3 Å of the ligand in the bound state (defined as binding site residues).

JAK-inhibitor complex	Interacting Residues
JAK2-inhibitor 5	Leu855, Gly856, Lys857, Val863, Ala880, Val911, Met929, Glu930, Tyr931, Leu932, Pro933, Gly935, Ser936, Arg980, Asn981, Leu983, Gly993
JAK2-inhibitor 6	Leu855, Gly856, Lys857, Val863, Ala880, Met929, Glu930, Tyr931, Leu932, Pro933, Gly935, Ser936, Arg980, Asn981, Ile982, Leu983, Gly993
JAK2-inhibitor 7	Gln853, Leu855, Val863, Ala880, Met929, Glu930, Tyr931, Leu932, Pro933, Gly935, Ser936, Leu983, Gly993, Asp994
JAK2-inhibitor 9	Leu855, Gly856, Lys857, Val863, Ala880, Val911, Met929, Glu930, Tyr931, Leu932, Pro933, Tyr934, Gly935, Ser936, Leu983, Gly993
JAK3-inhibitor 5	Leu828, Gly829, Val836, Ala853, Val884, Met902, Glu903, Tyr904, Leu905, Pro906, Gly908, Cys909, Arg953, Asn954, Leu956, Ala966
JAK3-inhibitor 6	Leu828, Val836, Ala853, Val884, Met902, Glu903, Tyr904, Leu905, Pro906, Gly908, Cys909, Arg916, Arg953, Asn954, Leu956, Ala966, Asp967
JAK3-inhibitor 7	Leu828, Gly829, Val836, Ala853, Val884, Met902, Glu903, Tyr904, Leu905, Pro906, Gly908, Cys909, Arg953, Asn954, Leu956, Ala966, Asp967
JAK3-inhibitor 9	Leu828, Gly829, Val836, Ala853, Val884, Met902, Glu903, Tyr904, Leu905, Pro906, Gly908, Cys909, Arg953, Asn954, Leu956, Asp967

Table S2: Experimentally determined vs. the SEEKR2 calculated residence times of inhibitors with the kinase domain of the JAK2 and JAK3 proteins. The SEEKR2 residence times and error estimates are the mean of each of the three SEEKR2 simulations.

JAK-inhibitor complex	Experimental Residence Time (hrs.)	SEEKR2 Residence Time (hrs.)
JAK2-inhibitor 5	2.68	1.70 ± 0.004
JAK2-inhibitor 6	6.65	6.79 ± 0.012
JAK2-inhibitor 7	3.20	2.31 ± 0.007
JAK2-inhibitor 9	11.07	10.96 ± 0.096
JAK3-inhibitor 5	0.98	0.54 ± 0.003
JAK3-inhibitor 6	0.78	0.19 ± 0.001
JAK3-inhibitor 7	1.30	1.12 ± 0.006
JAK3-inhibitor 9	3.15	2.53 ± 0.011

Table S3: Unpaired t-test to measure the statistical significance of the difference between the SEEKR2 calculated residence times of inhibitors with the kinase domain of the JAK2 and JAK3 proteins with the experimentally determined residence times.

JAK-inhibitor complex	SEEKR2 Mean Residence Time (hrs.)	Experimental Residence Time (hrs.)	t-ratio	p-value	Statistically Significant Difference (α=0.05)
JAK2-inhibitor 5	1.702	2.68	1.132	0.375148	No
JAK2-inhibitor 6	6.792	6.65	0.1065	0.924872	No
JAK2-inhibitor 7	2.306	3.20	1.082	0.392391	No
JAK2-inhibitor 9	10.960	11.07	0.0177	0.987493	No
JAK3-inhibitor 5	0.5379	0.98	1.26	0.334802	No
JAK3-inhibitor 6	0.1872	0.78	3.597	0.069358	No
JAK3-inhibitor 7	1.116	1.30	0.119	0.916129	No
JAK3-inhibitor 9	2.535	3.15	0.3385	0.767246	No

Table S4: Residues of JAK proteins interacting with inhibitor 9 obtained from the minimum average distance analysis within a cut-off distance of 4 Å averaged over three independent 2 μs MD simulation trajectories.

JAK-inhibitor complex	Interacting Residues
JAK2-inhibitor 9	Gln853, Leu855, Gly856, Lys857, Val863, Ala880, Val911, Met929, Glu930, Tyr931, Leu932, Pro933, Tyr934, Gly935, Ser936, Arg980, Asn981, Ile982, Leu983, Gly993, Asp994
JAK3-inhibitor 9	Leu828, Gly829, Val836, Ala853, Val884, Met902, Glu903, Tyr904, Leu905, Pro906, Ser907, Gly908, Cys909, Arg953, Asn954, Ile955, Leu956, Ala966, Asp967

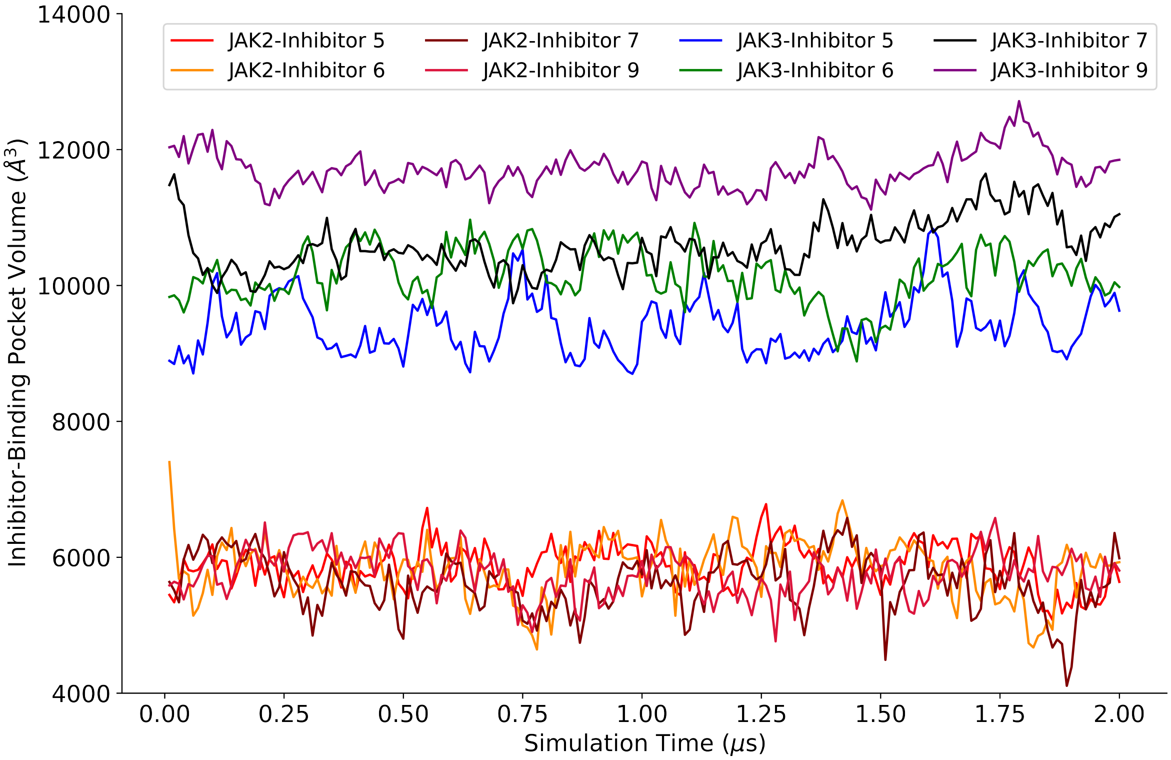

Figure S1: Pocket volume analysis for all the JAK2 and JAK3 inhibitor complexes.

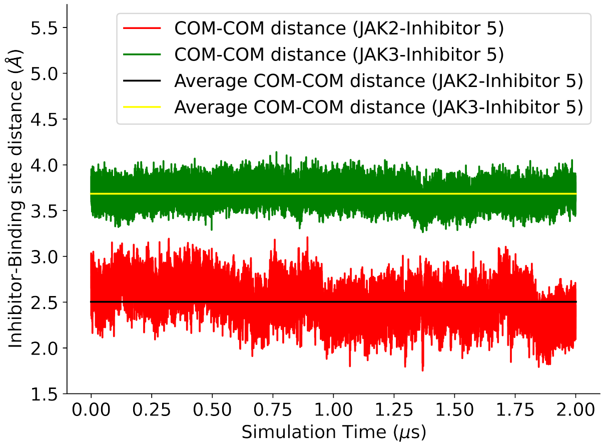

(a)

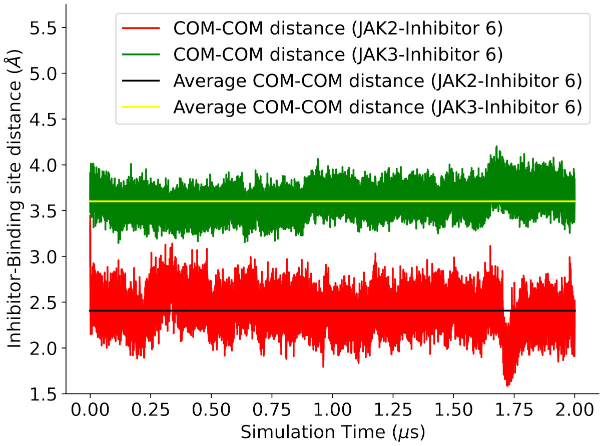

(b)

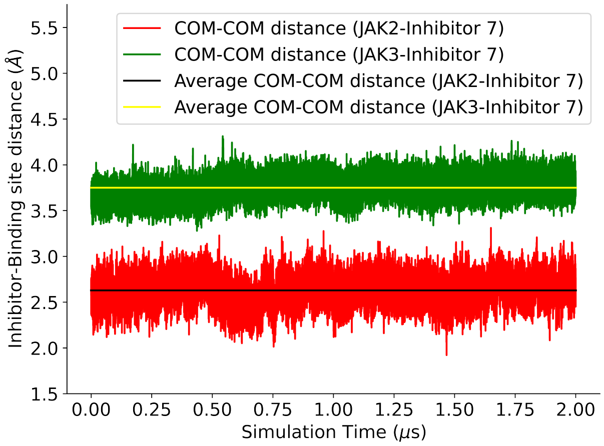

(c)

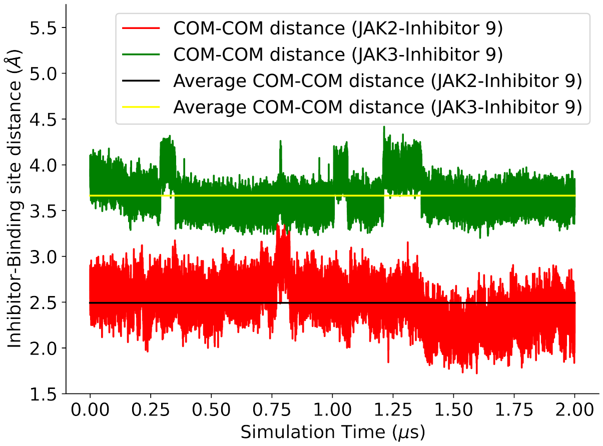

(d)

Figure S2: Inhibitor-Binding site distance analysis for JAK2 and JAK3 inhibitor complexes from three independent 2 μs MD simulation trajectories. The distance between the center of masses of the inhibitors and the α-C atoms of the binding site are used to calculate the inhibitor-binding site distance. (b) JAK2-inhibitor 5 vs. JAK3-inhibitor 5 complex (c) JAK2-inhibitor 6 vs. JAK3-inhibitor 6 complex (d) JAK2-inhibitor 7 vs. JAK3-inhibitor 7 complex (e) JAK2-inhibitor 9 vs. JAK3-inhibitor 9 complex
